# Key Factors Identification and Risk Assessment for the Stability of Deep Surrounding Rock in Coal Roadway

**DOI:** 10.3390/ijerph16152802

**Published:** 2019-08-06

**Authors:** Dongmei Huang, Weijun Li, Xikun Chang, Yunliang Tan

**Affiliations:** 1 State Key Laboratory of Mining Disaster Prevention and Control Co-Founded by Shandong Province and the Ministry of Science and Technology, Shandong University of Science and Technology, Qingdao 266590, China; 2 College of Mining and Safety Engineering, Shandong University of Science and Technology, Qingdao 266590, China

**Keywords:** key factors, risk assessment, deep surrounding rock stability, AHP-GRA

## Abstract

In order to evaluate the stability of deep surrounding rock, all of the affecting factors should be theoretically identified. However, some factors have slight impacts on the stability of deep surrounding rock compared with others. To conduct an effective risk assessment, key factors should be first extracted. The analytic hierarchy process (AHP) and grey relation analysis (GRA) methods are integrated to determine the key factors. First, the AHP method is applied to sort the factors by calculating the weights of them. Seven out of fifteen factors are extracted as the key factors, which account for 80% of the weights. Further, the GCA method is used to validate the effects of these key factors by analyzing the correlation between the performance of each factor and that of the reference. Considering the influence of these key factors and experts’ judgements, the multilevel fuzzy comprehensive evaluation method is adopted to obtain the risk level of the deep surrounding rock stability. Finally, the risk assessment of the deep surrounding rock in the E-Zhuang coal mine of Chinese Xinwen Mining Area illustrates the operability of the proposed method.

## 1. Introduction

The geological environment of coal mine is complex and its ecological environment is fragile. In recent years, the conditions of coal mines in China have gradually developed into deep and complex ones. The depth and breadth of mining have been increasing and rock engineering has been more difficult. Meanwhile, the gradual enlargement of the mining scale and the continuous improvement of the mechanization level have brought great difficulties to the stability control of surrounding rock in mines. In addition, factors such as stress and mining influence have led to the damage of weak rock surfaces in the process of excavation and artificial disturbance. Once the whole rock mass is destroyed, the instability of the surrounding rock could lead to a series of environmental disasters, such as collapse and roof separation, which result in great harm to the geological environment. From 2013 to 2017, nearly 39% of coal mine accidents in China were caused by roof accidents. The particular environment of the mine and these disasters indicate that the stability of the deep surrounding rock of the coal roadway is an urgent problem for safe mining. Therefore, it is of great significance to evaluate the stability of deep surrounding rock, which is not only related to the safety of people’s lives and property, but also to an urgent problem of safety in mining and environmental protection.

Deep surrounding rock plays an important role in the coal mining process. The instability of the deep surrounding rock will result in accidents such as roof caving and wall caving. During the years 2013–2017, nearly 39% of coal mining fatalities in China were caused by roof accidents. Substantial disasters have indicated that the stability of the deep surrounding rock in the coal roadway is an urgent problem for safe mining. Therefore, it is significant to conduct the evaluation of the deep surrounding rock stability.

Many works on surrounding rock stability evaluation are now available. For example, Kentil et al [1] studied the engineering geological properties of exposed rocks on the Ankara-Pozanti Expressway and evaluated the stability of excavated slopes. Andonov [2] evaluated the geomechanical conditions for establishing a free channel irrigation system and proposed some suggestions for improving the stability of rock mass. Saffari et al [3] applied a rock engineering systems approach to evaluate and classify the coal spontaneous combustion potential in the Eastern Alborz coal mines. Wang et al [4] used the ideal point method to categorize the affecting indices of surrounding rock stability and combined the objective and subjective weighting method to obtain the grade of the surrounding rock. Mishra et al [5] proposed the geotechnical risk management concept and applied it to the intelligent deep mines; Galvin [6] emphasized the importance of risk management in ground engineering and provided many management strategies. Babaeian et al [7] proposed a new framework for rock evaluation in open pit mines, integrating the multivariate regression method and the decision-making trial. Santos et al [8] proposed a method to predict the stability of the mine rock slope based on the principal component analysis and discriminant analysis. It can be seen that the stability of surrounding rock is determined by multiple indices and there is no consensus on stability affecting factors.

In fact, the stability of the deep surrounding rock is affected by not only geological features, but environmental factors and engineering factors. Theoretically, all of these affecting factors should be considered for a comprehensive risk assessment of the deep surrounding rock stability. However, it could be time-consuming to include all of the affecting factors in the assessment model. In fact, some of these factors only have a small impact on the stability of the deep surrounding rock. Therefore, a scientific method is required to extract those with a high important degree. One efficient method is to rank these factors and choose the top-ranked ones [9,10,11]. The analytic hierarchy process (AHP) has been proven to be a useful method for selecting indicators [12,13,14,15,16,17,18], but the relative importance between every two factors in AHP is determined by subjective judgment. There lacks the verification of these affecting factors. On the other hand, with the complexity of affecting factors, it is impracticable to obtain a definite value to express the stability of the deep surrounding rock, especially in situations where many of these factors cannot be measured precisely and subjective judgment from experts is inevitable. Therefore, a method to synthesize the key affecting factor and the experts’ knowledge is required. 

The novelty of this paper is to identify and verify the key factors of the deep surrounding rock stability and to establish a model for a comprehensive risk assessment. The rest of this paper is organized as follows: Section 2 introduces the key factors identification method and a fuzzy risk assessment method. Section 3 is the application of these methods with the E-Zhuang coal mine of Chinese Xinwen Mining Area as a case study. Section 4 provides conclusions.

## 2. Methodology

### 2.1. Key Factors Identification Method

Theoretically, all of the affecting factors should be identified to evaluate the stability of the deep surrounding rock, but with the limits of time and resource, only those contributing significantly to the stability of the deep surrounding rock can be considered. We named these factors as key factors. On the other hand, different affecting factors of the stability of the deep surrounding rock have different dimensions. Besides, some factors such as the seepage quantity of groundwater can be quantified, while others such as the purposes and age limit of the roadway cannot. All of these features of the affecting factors of the deep surrounding rock stability result in the inadaptability of any mathematical function. Therefore, experts’ knowledge and experience should be considered in a good way. 

**Figure 1:**
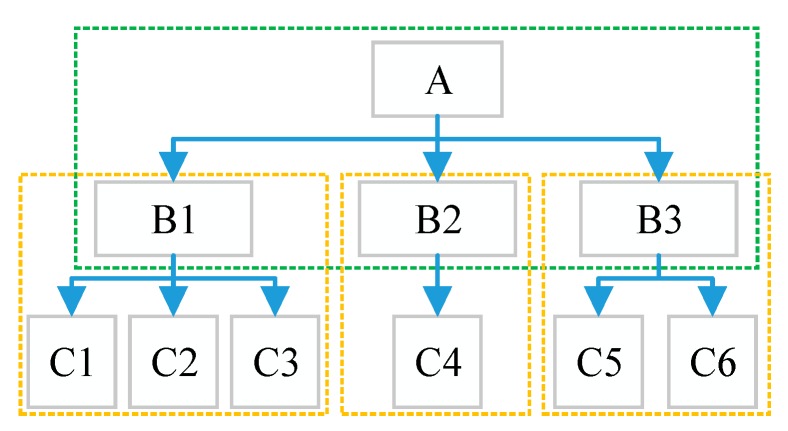
The unit division in a three-layered structure.

In order to determine the key factors contributing significantly to the stability of the deep surrounding rock in the coal roadway, an integrated method combining the AHP and GCA is proposed. The principle of the integrated method is shown as follows:

(1) Identify all the affecting factors (*F_i_*, *i* = 1, 2, …, *n*) and construct a hierarchy structure. The analytical object is named the target (top) layer. Then the categories of factors are classified in the criterion (middle) layer and all of the affecting factors compose the index (bottom) layer. 

(2) Divide units for the hierarchy structure. Each factor in the upper layer and its affecting factors are included in one unit. For example, there is a three-layered structure, and the unit division is shown in Figure 1.

(3) Represent the relative importance of different factors in each unit with the numbers 1–9 or the multiplicative inverse. Then the judgment matrices are determined. An example is shown in Equation (1). Assume *B* is in the criterion layer. *C*_1_, *C*_2_, and *C*_3_ are the affecting factors of *B*, which are in the index layer. According to expert judgement, *C*_2_ is relatively more important than C_1_ but less important than *C*_3_, and *C*_3_ is much more important than *C*_1_. Then the value assigned to *C*_3_ is bigger than the value of *C*_2_ and the value assigned to *C*_2_ is bigger than the value of *C*_1_. Note that the relative value determines the relative importance of different factors. The consistency of each judgment matrix should be checked, and the details can be seen in reference [19].

(1) $$R=\begin{array}{c} \begin{array}{cccc} B & \;C_{1} & \;C_{2} & \;C_{3} \end{array}\\ \begin{array}{cc} \begin{array}{c} C_{1}\\ C_{2}\\ C_{3} \end{array} & \left[\begin{array}{ccc} 1 & 2 & 3\\ 1/2 & 1 & 1/2\\ 1/3 & 2 & 1 \end{array}\right] \end{array} \end{array}$$

(4) Calculate the maximum characteristic root and the feature vector. In MATLAB, we can obtain the characteristic roots and the feature vector with the function *eig*, i.e., [*x*, *y*] = *eig*(*A*). The feature vector to which the maximum characteristic root corresponds is the weight vector. Further, the weight vector should be normalized. 

(5) Calculate the combination weights. With the judgment matrices, we can only get the weight of each factor in each individual unit. The objective is to obtain the weight of each factor in the index layer for the target layer. Therefore, the weights should be combined. Assume that the weight vectors in each unit shown in Figure 1 are represented as *W_C_*_→*B*1_, *W_C_*_→*B*2_, *W_C_*_→*B*3_, *W_B_*_→*A*_, then the combination weights can be calculated as:

(2) $$W_{C\to B1\to A}=W_{B\to A}\times W_{C\to B1}$$

(3) $$W_{C\to B1\to A}=W_{B\to A}\times W_{C\to B1}$$

(4) $$W_{C\to B1\to A}=W_{B\to A}\times W_{C\to B1}$$

(6) Sort the affecting factors according to their weights and extract the key factors, which account for 80% of the weights. 

(7) Validate the results of AHP with GRA. The correlation between the measured value and reference value can be reflected with GRA (Grey Relation Analysis). Accordingly, the measured value of the key factors should be highly correlated with the reference value. To verify, the indicators reflecting the measured value and the reference value should be first defined. 

① Define the correlation indicators and sequences.

For the affecting factors of the deep surrounding rock stability, the indicators include the possibility of accidents, the time of exposure to hazardous conditions, consequence, and current control measures. The grading standards of these indicators are defined as shown in Table 1. The score is determined by considering the comprehensive correlation factors in the MLS method. MLS is one of the risk assessment methods for the working conditions. The control correlation strength indices of the surrounding rock stability of the deep roadway factors are chosen, which separately are: damage possibility (accident possibility), cycle strength (exposure time to dangerous environment), disaster degree (possible consequence), and control measures (existing prevention and control measures). The value range of the four indices is also determined by this method, and then the correlation coefficient and correlation degree are calculated using MATLAB. Here the relevance degree is a comprehensive representation of the importance level of each factor. The smaller the degree, the greater the importance.

For each affecting factor, there is a parameter sequence consisting of these four indicators, which is *X_i_* = {value 1 (possibility of accidents), value 2 (time of exposure to hazardous conditions), value 3 (consequence), and value 4 (current control measures)}. In addition, a reference sequence is defined as *X*_0_ = {0.1, 0.5, 1, 1} based on the optimum value of each indicator.

② Calculate the correlation coefficient.

Assume that $X_{0}=\{x_{0}(t_{k}),k=1,2,\cdots ,n\}$ represents the reference sequence and $X_{i}=\{x_{i}(t_{k}),k=1,2,\cdots ,n\}$ represents the parameter sequence. Calculate the absolute difference ${>}_{i}\left(n\right)$ between the value of each indicator $x_{i}(n)$ and the reference value $x_{0}(n)$ [20,21]. The equation is as follows:

(5) $${>}_{i}\left(n\right)=\left|x_{0}(n)-x_{i}(n)\right|$$

Then the correlation coefficient $\xi_{i}(n)$ can be calculated based on Equation (6):

(6) $$\xi_{i}\left(n\right)=\frac{{>}_{\min}+\rho {>}_{\max}}{{>}_{i}\left(n\right)+\rho {>}_{\max}}=\frac{\underset{i}{\min}\left|x_{0}-x_{i}\right|+\rho \underset{i}{\min}\left|x_{0}-x_{i}\right|}{\left|x_{0}-x_{i}\right|+\rho \underset{i}{\max}\left|x_{0}-x_{i}\right|},\;n=1,2,3,4;\;i=1,2,\cdots ,n$$

The smaller that *ρ* is, the bigger the correlation coefficient difference. In general, ρ=0.5.

③ Calculate the correlation degree.

Since there are four indicators for each factor, the correlation degree should reflect all of these four aspects. Therefore, the mean value of correlation coefficients is defined as the correlation degree. The smaller the correlation degree, the more dangerous is the affecting factor. The equation is shown as follows:

(7) $$\gamma_{i}=\frac{1}{n}\sum_{k=1}^{n}\xi_{i}(k)$$

### 2.2. Fuzzy Risk Assessment Method

The risk of the deep surrounding rock stability can be divided into five levels. The risk evaluation set is shown as follows:

*V* = (*V*_1_, *V*_2_, *V*_3_, *V*_4_, *V*_5_) = {very low, relatively low, general, relatively high, very high} (8)

(1) Establish the risk assessment matrix ${\left(M_{ij}\right)}_{k\times m}$. *K* experts are invited to evaluate the key factors of the deep surrounding rock stability. The matrix ${\left(M_{ij}\right)}_{k\times m}$ is shown as follows:
  (9) $${\left(M_{ij}\right)}_{k\times m}=\left(\begin{array}{cccc} M_{11} & M_{12} & \cdots & M_{1m}\\ M_{21} & M_{22} & \cdots & M_{2m}\\ \vdots & \vdots & \ddots & \vdots \\ M_{k1} & M_{k2} & \cdots & M_{km} \end{array}\right)$$
(2) Determine the membership matrix.

Segmented grey whitening weight functions are used to transform the evaluation value into membership degrees. Therefore, the evaluation value is the independent variable, and the memberships are the dependent variables. In response to five risk evaluation levels, five-segmented functions are defined.

(1) The grey whitening weight function is shown in Equation (10) when the risk evaluation level is “very high”:

(10) $$f_{1}(d_{i})=\{\begin{array}{c} 0\\ d_{i}/5\\ 1 \end{array}\;\begin{array}{c} d\notin \left[0,10\right]\\ d\in \left[0,5\right]\\ d\in \left[5,10\right] \end{array}$$

(2) The grey whitening weight function is shown in Equation (11) when the risk evaluation level is “relatively high”:

(11) $$f_{2}(d_{i})=\{\begin{array}{c} 0\\ d_{i}/4\\ \left(8-d_{i}\right)/4 \end{array}\;\begin{array}{c} d\notin \left[0,8\right]\\ d\in \left[0,4\right]\\ d\in \left[4,8\right] \end{array}$$

(3) The grey whitening weight function is shown in Equation (12) when the risk evaluation level is “general”:

(12) $$f_{3}(d_{i})=\{\begin{array}{c} 0\\ d_{i}/3\\ \left(6-d_{i}\right)/3 \end{array}\;\begin{array}{c} d\notin \left[0,6\right]\\ d\in \left[0,3\right]\\ d\in \left[3,6\right] \end{array}$$

(4) The grey whitening weight function is shown in Equation (13) when the risk evaluation level is “relatively low”:

(13) $$f_{4}(d_{i})=\{\begin{array}{c} 0\\ d_{i}/2\\ \left(4-d_{i}\right)/2 \end{array}\;\begin{array}{c} d\notin \left[0,4\right]\\ d\in \left[0,2\right]\\ d\in \left[2,4\right] \end{array}$$

(5) The grey whitening weight function is shown in Equation (14) when the risk evaluation level is “very low”:

(14) $$f_{5}(d_{i})=\{\begin{array}{c} 0\\ 1\\ 2-d_{i} \end{array}\;\begin{array}{c} d\notin \left[0,2\right]\\ d\in \left[0,1\right]\\ d\in \left[1,2\right] \end{array}$$

Then after normalization, each key factor’s membership degree to each risk level can be obtained with the grey whitening weight functions. For the factor *j*, its membership degree to risk level *e* can be represented with Equation (15):

(15) $$s_{e}=\frac{\sum_{i=1}^{k}f_{e}\left(M_{ij}\right)}{\sum_{e=1}^{5}\sum_{i=1}^{k}f_{e}\left(M_{ij}\right)}$$

where *i* refers to the experts and *i* = 1, 2, …, *k*; *j* represents the factor and *j* = 1, 2, …, *m*; *e* means the risk level; and *e* = 1, 2, 3, 4, 5. For factor *j*, the membership degree vector is shown below:

(16) $$S_{11}=\left(s_{111},s_{112},s_{113},s_{114},s_{115}\right)$$

Similarly, the membership degree vectors of other factors can be obtained. Then the membership matrix is shown in Equation (17):

(17) $$S=\left(\begin{array}{c} S_{11}\\ S_{12}\\ \vdots \\ S_{ij} \end{array}\right)\left(\begin{array}{cccc} s_{111} & s_{112} & \cdots & s_{115}\\ s_{121} & s_{122} & \cdots & s_{125}\\ \vdots & \vdots & \ddots & \vdots \\ s_{ij1} & s_{ij2} & \cdots & s_{ij5} \end{array}\right)$$

(3) Multi-level fuzzy comprehensive evaluation.

To evaluate a system with affecting factors interrelated in a hierarchy structure, the multi-level fuzzy comprehensive evaluation has proven to be an effective method [22,23]. The membership degree of each factor in the lower level to its corresponding upper level factor can be obtained with a fuzzy comprehensive evaluation [24,25,26]. The weight set consists of weights of key factors, represented as {a,a,a}. By integrating the weight set with membership matrix through a fuzzy operator, the fuzzy comprehensive evaluation results are available. For a three-layered structure, the fuzzy comprehensive evaluation should be conducted twice. First, calculate the membership degrees of one factor in the intermediate layer to each risk level. The equation is as follows:

(18) $$\begin{array}{l} B_{i}=A_{i}\cdot S_{i}=\left(a_{i1},a_{i2},\cdots a_{ij}\right)\left(\begin{array}{ccc} s_{i11} & \cdots & s_{i1k}\\ \vdots & \ddots & \vdots \\ s_{ij1} & \cdots & s_{ijk} \end{array}\right)\\ \;=\left(b_{i1},b_{i2},\cdots b_{ik}\right) \end{array}$$

Similarly, the membership degrees of other factors in the intermediate layer to each risk level can be obtained, and all these membership degree sets form a membership degree matrix:

(19) $$S=\left(\begin{array}{c} B_{1}\\ B_{2}\\ \vdots \\ B_{m} \end{array}\right)\left(\begin{array}{c} A_{1}\cdot S_{1}\\ A_{2}\cdot S_{2}\\ \vdots \\ A_{m}\cdot S_{m} \end{array}\right)$$

Then the risk level of the object can be calculated according to Equation (20):

(20) $$\begin{array}{l} B=A\cdot S=\left(a_{1},a_{2},\cdots ,a_{m}\right)\left(\begin{array}{ccc} b_{11} & \cdots & b_{1k}\\ \vdots & \ddots & \vdots \\ b_{m1} & \cdots & b_{mk} \end{array}\right)\\ \;=\left(b_{1},b_{2},\cdots ,b_{k}\right) \end{array}$$

## 3. Applications

### 3.1. Affecting Factors of Deep Surrounding Rock Stability

**Figure 2:**
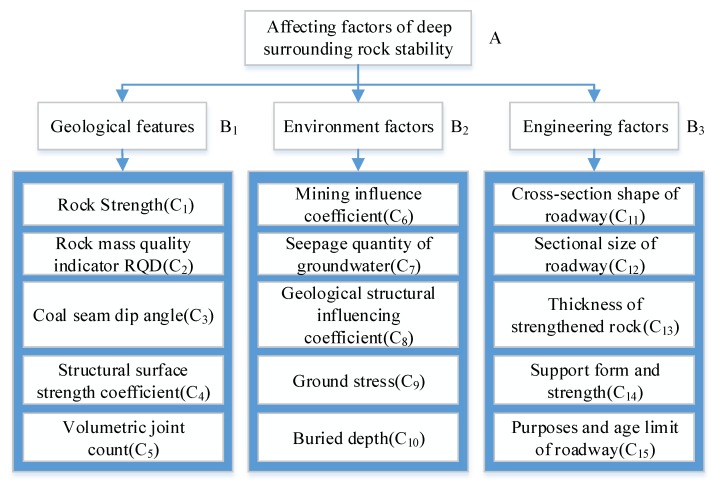
Hierarchy structure model for the deep surrounding rock stability affecting factors.

The stability of the deep surrounding rock is affected by geological features such as rock strength, environmental factors such as ground stress, and engineering factors such as the thickness of strengthened rock [27,28,29]. To evaluate the risk of the deep surrounding rock, all these aspects should be considered. Based on field experience and experts’ knowledge, 15 affecting factors are identified for the risk evaluation of an E-Zhuang coal mine in Chinese Xinwen Mining Area. The hierarchy structure model for the deep surrounding rock stability affecting factors is shown in Figure 2. However, some of them have little impact on the stability of the deep surrounding rock. The methods introduced above are applied to extract the key ones.

### 3.2. Key Factors Identification Based on AHP and GCA

#### 3.2.1. Determine the Weights of Each Factor with AHP

According to AHP, pairwise comparisons are conducted for the 15 affecting factors. The scoring results are shown in Table 2.

According to the calculation rules introduced in Section 2.1, the weights of each factor can be calculated. The analysis and ranking results are shown in Table 3. The bigger the weight, the more important the factor. It can be seen that the top 7 factors’ weights account for 0.8151, which means that these 7 factors are key factors for the deep surrounding rock stability. 

The results are in consistent with the actual conditions of the deep surrounding rock in the coal roadway. In fact, the rock strength, rock mass quality indicator RQD, and coal seam dip angle are the direct causes of the deep surrounding rock failure according to accident statistics. The mining influence coefficient and ground stress are the precipitating factors because they determine the environment of the surrounding rock. The sectional size of the roadway and the thickness of the strengthened rock are also important contributors since great uncertainties exist during engineering excavation. 

#### 3.2.2. Validate the Results of AHP with GRA

GRA (Grey Relation Analysis) extracts the key factors by calculating the correlation between the measured value and reference value. Four correlation indicators have been defined in Section 2.1, which are the possibility of accidents, the time of exposure to hazardous conditions, consequence, and current control measures. According to the grading standards of these indicators presented in Table 1, experts in the E-Zhuang coal mine of Chinese Xinwen Mining Area assigned evaluation values for each factor. Accordingly, there are 15 parameter sequences and one reference sequence, shown in Table 4, and corresponding normalized data are shown in Table 5.

According to Equations (5)–(8) mentioned in Section 2.1, the correlation coefficients and correlation degrees for each factor can be calculated, shown in Table 6. Then these 15 factors are sorted by their correlation degrees. 

From Table 5 we can conclude that the rock strength, rock mass quality indicator RQD, coal seam dip angle, mining influence coefficient, ground stress, sectional size of the roadway, and thickness of strengthened rock are dominant factors of the deep surrounding rock stability in the coal roadway. The results validate the results of AHP. 

### 3.3. Fuzzy Risk Evaluation

(1) Establish the evaluation index system.

**Figure 3:**
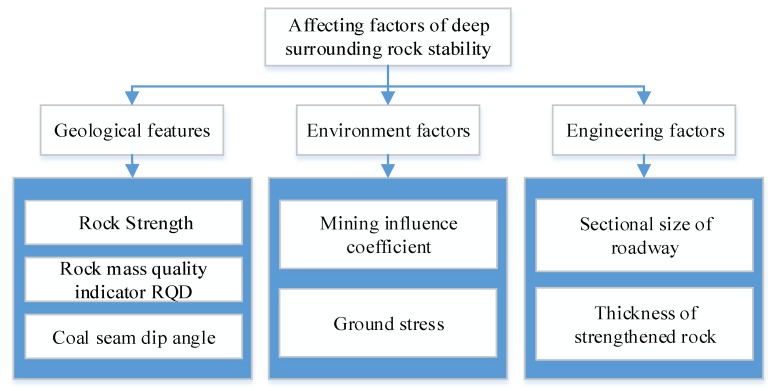
Deep surrounding rock stability evaluation index system.

According to the key factors identification results, a structural evaluation index system can be established, shown in Figure 3. Based on the index system, two layers of fuzzy comprehensive evaluation are defined. The first layer is composed of the bottom factors and the intermediate indices, and the second layer consists of the intermediate indices and the top object. The evaluation factor set for the second layer is represented as $T=\left\{T_{1},T_{2},T_{3}\right\}$, and the evaluation factor set for the first layer is represented as $M_{1}=\left\{M_{11},M_{12},M_{13}\right\}$, $M_{2}=\left\{M_{21},M_{22},M_{23}\right\}$ and $M_{3}=\left\{M_{31},M_{32},M_{33}\right\}$.

(2) Establish the weight set.

According to the AHP results, the weights of these seven key factors are available. Further, the normalized weights of them are calculated, as shown in Table 7. Take rock strength as an example; the calculation process is as follows:

(21) $$\mathrm{Normalized}\;\mathrm{weight}\;(\mathrm{rock}\;\mathrm{strength})=\frac{0.2948}{0.2948+0.1150+0.0761}=0.6067$$

Accordingly, the weight sets are represented as follows:

(22) $$C_{1}=(0.6067\;0.2367\;0.1566)$$

(23) $$C_{2}=(0.5068\;0.4932)$$

(24) $$C_{3}=(0.6188\;0.3812)$$

(3) Determine the membership matrix.

Further, 10 experts were employed to evaluate these seven key factors. For each key factor, every expert assigns a number between 0 and 10 according to the interval definition for each risk evaluation level. 

The risk of the deep surrounding rock stability can be divided into five levels. The risk evaluation set is *V* = (*V*_1_, *V*_2_, *V*_3_, *V*_4_, *V*_5_) = {very low, relatively low, general, relatively high, very high}. Then the evaluation matrix ${\left(M_{ij}\right)}_{10\times 7}$ of these seven factors is obtained, shown in Equation (25), where $M_{ij}$ represents the risk evaluation value of factor *j* made by expert *i*, and where *i* refers to the experts and *i* = 1, 2, …, *k*; *j* represents the factor and *j* = 1, 2, …, *m*:

(25) $${\left(M_{ij}\right)}_{10\times 7}=\left(\begin{array}{cccc} M_{11} & M_{12} & \cdots & M_{1,7}\\ M_{21} & M_{22} & \cdots & M_{2,7}\\ \vdots & \vdots & \ddots & \vdots \\ M_{10,1} & M_{10,2} & \cdots & M_{10,7} \end{array}\right)=\left[\begin{array}{ccccccc} 4 & 2 & 2 & 6 & 7 & 6 & 4\\ 6 & 3 & 3 & 7 & 8 & 5 & 5\\ 5 & 2 & 2 & 6 & 7 & 4 & 4\\ 4 & 3 & 3 & 6 & 6 & 5 & 5\\ 5 & 2 & 4 & 7 & 7 & 4 & 4\\ 4 & 4 & 4 & 6 & 8 & 4 & 6\\ 6 & 3 & 2 & 8 & 6 & 6 & 5\\ 5 & 3 & 3 & 6 & 7 & 5 & 4\\ 4 & 4 & 4 & 7 & 7 & 4 & 5\\ 5 & 2 & 2 & 6 & 8 & 6 & 6 \end{array}\right]$$

For example, for the factor *j*, its membership to risk evaluation level “very low” can be calculated as:

(26) $$\begin{array}{l} s_{1}=\frac{\sum_{i=1}^{k}f_{1}\left(M_{i1}\right)}{\sum_{e=1}^{5}\sum_{i=1}^{k}f_{e}\left(M_{i1}\right)}=\frac{\sum_{i=1}^{10}f_{1}\left(M_{i1}\right)}{\sum_{e=1}^{5}\sum_{i=1}^{10}f_{e}\left(M_{i1}\right)}\\ \;=\frac{f_{1}\left(4\right)+f_{1}\left(6\right)+f_{1}\left(5\right)+f_{1}\left(4\right)+f_{1}\left(5\right)+f_{1}\left(4\right)+f_{1}\left(6\right)+f_{1}\left(5\right)+f_{1}\left(4\right)+f_{1}\left(5\right)}{\sum_{i=1}^{10}f_{1}\left(M_{i1}\right)+\sum_{i=1}^{10}f_{2}\left(M_{i1}\right)+\sum_{i=1}^{10}f_{3}\left(M_{i1}\right)+\sum_{i=1}^{10}f_{4}\left(M_{i1}\right)+\sum_{i=1}^{10}f_{4}\left(M_{i1}\right)}\\ \;=\frac{9.2}{9.2+8.0+4.0+0+0}\\ \;=0.4340 \end{array}$$

Similarly, the memberships of factor *j* to other risk evaluation levels can be calculated. Consequently, the membership degree set of factor *j* is shown below:

(27) $$S_{11}=\left[\begin{array}{ccccc} 0.4340 & 0.3774 & 0.1887 & 0.0000 & 0.0000 \end{array}\right]$$

The other factors’ membership sets can be established and represented as:

(28) $$S_{1}=\left[\begin{array}{ccccc} 0.4340 & 0.3774 & 0.1887 & 0.0000 & 0.0000\\ 0.2105 & 0.2632 & 0.3008 & 0.2256 & 0.0000\\ 0.2212 & 0.2765 & 0.2925 & 0.2098 & 0.0000 \end{array}\right]$$

(29) $$S_{2}=\left[\begin{array}{ccccc} 0.7273 & 0.2727 & 0.0000 & 0.0000 & 0.0000\\ 0.8163 & 0.1837 & 0.0000 & 0.0000 & 0.0000 \end{array}\right]$$

(30) $$S_{3}=\left[\begin{array}{ccccc} 0.4462 & 0.3758 & 0.1780 & 0.0000 & 0.0000\\ 0.4340 & 0.3774 & 0.1887 & 0.0000 & 0.0000 \end{array}\right]$$

*S*_1_ represents the risk evaluation memberships of three key factors that reflect geological features to risk levels. *S*_2_ represents the risk evaluation memberships of two key factors that reflect environmental features to risk levels. *S*_3_ represents the risk evaluation memberships of three key factors that reflect engineering features to risk levels. 

(4) First layer fuzzy comprehensive evaluation.

According to the comprehensive evaluation principles, the first level risk evaluation of geological features (*B*_1_), environmental features (*B*_2_), and engineering features (*B*_3_) can be available. The calculation process is shown below:

(31) $$\begin{array}{l} B_{1}=C_{1}S_{1}=\left(\begin{array}{ccc} 0.6067 & 0.2367 & 0.1566 \end{array}\right)\left[\begin{array}{ccccc} 0.4340 & 0.3774 & 0.1887 & 0.0000 & 0.0000\\ 0.2105 & 0.2632 & 0.3008 & 0.2256 & 0.0000\\ 0.2212 & 0.2765 & 0.2925 & 0.2098 & 0.0000 \end{array}\right]\\ \;=\left(\begin{array}{ccccc} 0.3131 & 0.3012 & 0.2084 & 0.0777 & 0 \end{array}\right) \end{array}$$

(32) $$\begin{array}{l} B_{2}=C_{2}S_{2}=\left(\begin{array}{cc} 0.5068 & 0.4932 \end{array}\right)\left[\begin{array}{ccccc} 0.7273 & 0.2727 & 0.0000 & 0.0000 & 0.0000\\ 0.8163 & 0.1837 & 0.0000 & 0.0000 & 0.0000 \end{array}\right]\\ \;=\left(\begin{array}{ccccc} 0.5868 & 0.1741 & 0 & 0 & 0 \end{array}\right) \end{array}$$

(33) $$\begin{array}{l} B_{3}=C_{3}S_{3}=\left(\begin{array}{cc} 0.6188 & 0.3812 \end{array}\right)\left[\begin{array}{ccccc} 0.4462 & 0.3758 & 0.1780 & 0.0000 & 0.0000\\ 0.4340 & 0.3774 & 0.1887 & 0.0000 & 0.0000 \end{array}\right]\\ \;=\left(\begin{array}{ccccc} 0.3048 & 0.2598 & 0.1257 & 0 & 0 \end{array}\right) \end{array}$$

(5) Second layer fuzzy comprehensive evaluation.

The first level evaluation results (*B*_1_, *B*_2_, *B*_3_) are used as input data for the second layer fuzzy comprehensive evaluation. The calculating process is as follows:

(34) $$S=\left(\begin{array}{c} B_{1}\\ B_{2}\\ B_{3} \end{array}\right)\left(\begin{array}{c} C_{1}\cdot S_{1}\\ C_{2}\cdot S_{2}\\ C_{3}\cdot S_{3} \end{array}\right)=\left[\begin{array}{ccccc} 0.3131 & 0.3012 & 0.2084 & 0.0777 & 0\\ 0.5868 & 0.1741 & 0 & 0 & 0\\ 0.3048 & 0.2598 & 0.1257 & 0 & 0 \end{array}\right]$$

(35) $$\begin{array}{l} B=AS=\left(\begin{array}{ccc} 0.5396 & 0.1634 & 0.2970 \end{array}\right)\left[\begin{array}{ccccc} 0.3131 & 0.3012 & 0.2084 & 0.0777 & 0\\ 0.5868 & 0.1741 & 0 & 0 & 0\\ 0.3048 & 0.2598 & 0.1257 & 0 & 0 \end{array}\right]\\ \;=\left(\begin{array}{ccccc} 0.3554 & 0.2681 & 0.1498 & 0.0419 & 0 \end{array}\right) \end{array}$$

(6) Result analysis.

The calculation results indicate the risk of the deep surrounding rock stability. According to the maximum membership principle (Li et al., 2015), the risk level of the deep surrounding rock in the E-Zhuang coal mine of Chinese Xinwen Mining Area is very low and the membership degree is 0.3554. This case illustrates that the combined approach can help identify key factors of the deep surrounding rock stability and obtain the risk level.

## 4. Conclusions

(1) The first and most important step of risk evaluation is to identify risk affecting factors. The stability of the deep surrounding rock is affected not only by geological features, but also by environmental factors and engineering factors. Since not all of these factors can be measured or quantified, especially man-made ones, a semi-quantitative approach is necessary. In order to extract the key affecting factors and evaluate the risk level of the deep surrounding rock stability, a method combing AHP, GCA, and FCE is presented. 

(2) The traditional AHP has been a useful approach to obtain the weight of each factor. With GCA, the result of AHP can be verified. Therefore, the key affecting factors are identified, including rock strength, rock mass quality indicator RQD, coal seam dip angle, mining influence coefficient, ground stress, sectional size of roadway, and thickness of strengthened rock, which are dominant factors of the deep surrounding rock stability in the coal roadway. In addition, the weights of these main factors are calculated. 

(3) Further, a multilevel FCE method is used to include both the influence of these key affecting factors and the experts’ judgements. The comprehensive method was applied to the risk assessment of the deep surrounding rock in the E-Zhuang coal mine of Chinese Xinwen Mining Area to obtain the risk level. The results show that the risk level of the deep surrounding rock is extremely low and support measures are necessary. The results can also provide a reference for other similar deep surrounding rock.

(4) Although the stability of surrounding rock in the deep mine has been well controlled with the gradual improvement of underground rock engineering construction, the traditional geotechnical engineering still faces a series of new environmental protection problems. With the gradual development of coal mines in China to deeper and more complex conditions, the problems of environmental effects and ecological environment protection have not been paid enough attention. The rock mass stability evaluation can provide theoretical support for ecological environment protection.

## Figures and Tables

**Table 1:** The grading standards of correlation indicators.

Accident Possibility	Score	Time of Exposure	Score	Consequence	Score	Control Measures	Score
Absolutely impossible	0.1	Very rare	0.5	Minor consequences and no casualties	1	Preventive measures	1
Extremely unlikely	0.2	Several times per year	1	Relatively serious consequences and injuries	3	Emergency measures	3
Imaginable but highly unlikely	0.5	One time per month	2	Serious consequences and injuries	7	No control measures	5
Far less likely	1	One time per week or accidentally	3	Very serious and one death	15		
Not often but likely	3	Exposure to hazardous conditions every day	6	At least two deaths	40		
Very likely	6	Continual exposure to hazardous conditions	10	Multiple fatalities	100		
Predictable	10						

**Table 2:** Scoring table with AHP.

***A***		***B*_1_**		***B*_2_**		***B*_3_**	
*B*_1_		1		3		2	
*B*_2_		1/3		1		1/2	
*B*_3_		1/2		2		1	
***B*_1_**	***C*_1_**		***C*_2_**	***C*_3_**	***C*_4_**		***C*_5_**
*C*_1_	1		4	5	7		8
*C*_2_	1/4		1	2	4		6
*C*_3_	1/5		1/2	1	3		5
*C*_4_	1/7		1/4	1/3	1		2
*C*_5_	1/8		1/6	1/5	1/2		1
***B*_2_**	***C*_6_**		***C*_7_**	***C*_8_**	***C*_9_**		***C*_10_**
*C*_6_	1		4	5	1		7
*C*_7_	1/4		1	2	1/4		2
*C*_8_	1/5		1/2	1	1/5		2
*C*_9_	1		4	5	1		6
*C*_10_	1/7		1/2	1/2	1/6		1
***B*_3_**	***C*_11_**		***C*_12_**	***C*_13_**	***C*_14_**		***C*_15_**
*C*_11_	1		1/7	1/5	1/4		1/3
*C*_12_	7		1	2	3		4
*C*_13_	5		1/2	1	2		3
*C*_14_	4		1/3	1/2	1		2
*C*_15_	3		1/4	1/3	1/2		1

**Table 3:** Factors’ weights and ranks.

Factors	*B*_1_	*B*_2_	*B*_3_	*A*	Rank
*C*_1_	0.5463	-	-	0.2948	1
*C*_2_	0.2131	-	-	0.1150	3
*C*_3_	0.1410	-	-	0.0761	5
*C*_4_	0.0609	-	-	0.0329	9
*C*_5_	0.0387	-	-	0.0209	11
*C*_6_	-	0.3856	-	0.0630	6
*C*_7_	-	0.1103	-	0.0180	12
*C*_8_	-	0.0767	-	0.0125	14
*C*_9_	-	0.3753	-	0.0613	7
*C*_10_	-	0.0520	-	0.0085	15
*C*_11_	-	-	0.0461	0.0137	13
*C*_12_	-	-	0.4271	0.1268	2
*C*_13_	-	-	0.2631	0.0781	4
*C*_14_	-	-	0.1622	0.0482	8
*C*_15_	-	-	0.1015	0.0301	10

**Table 4:** Parameter sequence of each factor and reference sequence.

Factors	Accident Possibility	Time of Exposure	Consequence	Control Measures
Rock strength	10	10	100	3
Rock mass quality indicator RQD	6	6	40	5
Coal seam dip angle	3	6	15	5
Structural surface strength coefficient	1	3	1	3
Volumetric joint count	3	3	1	5
Mining influence coefficient	3	6	15	5
Seepage quantity of groundwater	1	1	7	1
Geological structural influencing coefficient	3	2	3	3
Ground stress	6	6	40	1
Buried depth	0.5	0.5	1	5
Cross-section shape of roadway	3	1	3	3
Sectional size of roadway	6	3	40	5
Thickness of strengthened rock	3	6	15	5
Support form and strength	3	6	7	3
Purposes and age limit of roadway	3	2	3	1
Reference value	0.1	0.5	1	1

**Table 5:** Parameter sequence and reference sequence after normalization.

Factors	Accident Possibility	Time of Exposure	Consequence	Control Measures
Rock strength	2.7523	2.4390	5.1546	0.8491
Rock mass quality indicator RQD	1.6514	1.4634	2.0619	1.4151
Coal seam dip angle	0.8257	1.4634	0.7732	1.4151
Structural surface strength coefficient	0.2752	0.7317	0.0516	0.8491
Volumetric joint count	0.8257	0.7317	0.0516	1.4151
Mining influence coefficient	0.8257	1.4634	0.7732	1.4151
Seepage quantity of groundwater	0.2752	0.2439	0.3608	0.2830
Geological structural influencing coefficient	0.8257	0.4878	0.1546	0.8491
Ground stress	1.6514	1.4634	2.0619	0.2830
Buried depth	0.1376	0.1220	0.0516	1.4151
Cross-section shape of roadway	0.8257	0.2439	0.1546	0.8491
Sectional size of roadway	1.6514	0.7317	2.0619	1.4151
Thickness of strengthened rock	0.8257	1.4634	0.7732	1.4151
Support form and strength	0.8257	1.4634	0.3608	0.8491
Purposes and age limit of roadway	0.8257	0.4878	0.1546	0.2830
Reference value	0.0275	0.1220	0.0516	0.2830

**Table 6:** Correlation coefficient and sorting of correlation degree.

Factors	Accident Possibility	Time of Exposure	Consequence	Control Measures	Correlation Degree	Rank
Rock strength	0.4836	0.5241	0.3333	0.8184	0.5399	1
Rock mass quality indicator RQD	0.6111	0.6554	0.5593	0.6927	0.6296	2
Coal seam dip angle	0.7617	0.6554	0.7795	0.6927	0.7223	7
Structural surface strength coefficient	0.9115	0.8707	1.0000	0.8184	0.8843	12
Volumetric joint count	0.7617	0.8071	1.0000	0.6927	0.8154	9
Mining influence coefficient	0.7617	0.6554	0.7795	0.6927	0.7223	7
Seepage quantity of groundwater	0.9115	0.9544	0.8919	1.0000	0.9394	15
Geological structural influencing coefficient	0.7617	0.8746	0.9612	0.8184	0.8540	10
Ground stress	0.6111	0.6554	0.5593	1.0000	0.7065	4
Buried depth	0.9586	1.0000	1.0000	0.6927	0.9128	14
Cross-section shape of roadway	0.7617	0.9544	0.9612	0.8184	0.8739	11
Sectional size of roadway	0.6111	0.8071	0.5593	0.6927	0.6676	3
Thickness of strengthened rock	0.7617	0.6554	0.7795	0.6927	0.7223	7
Support form and strength	0.7617	0.6554	0.8919	0.8184	0.7819	8
Purposes and age limit of roadway	0.7617	0.8746	0.9612	1.0000	0.8994	13

**Table 7:** The normalized weights of key factors.

Index Layer	Key Factors	Weights	Normalized Weights
Geological features	Rock strength	0.2948	0.6067
	Rock mass quality indicator RQD	0.1150	0.2367
	Coal seam dip angle	0.0761	0.1566
	Sum	0.4859	1
Environmental features	Mining influence coefficient	0.0630	0.5068
	Ground stress	0.0613	0.4932
	Sum	0.1243	1
Engineering features	Sectional size of roadway	0.1268	0.6188
	Thickness of strengthened rock	0.0781	0.3812
	Sum	0.2049	1
